# Effects of Non-pharmacological Interventions on Elderly in Nursing Homes with Sleep disorder: A Meta-Analysis

**DOI:** 10.1093/geroni/igab046.3385

**Published:** 2021-12-17

**Authors:** Sunok Jung, Eunju Choi, Hyeyoung Kim

**Affiliations:** College of Nursing, Ewha Womans University, Seoul, Seoul-t'ukpyolsi, Republic of Korea

## Abstract

Purpose: This study aimed to examine the effects of nonpharmacological sleep intervention programs to improve sleep quality among the elderly in long-term care facilities. Methods: A literature search and selection was performed on nine different databases using the Preferred Reporting Items for Systematic Review and Meta-Analysis Statement. In total, 14 studies met the inclusion criteria and were systematically reviewed. For the meta-analysis, the effect size was estimated using the random-effects model on Review Manager (RevMan) desktop version 5.4 of the Cochrane Library. Result: The meta-analysis of nonpharmacological interventions obtained a total effect size of 1.0 (standardized mean difference [SMD] = 1.0, 95% confidence interval [CI]: 0.64–1.35), which was statistically significant (Z = 5.55, p < .001). The most frequent nonpharmacological interventions identified were the interventions using aroma; the effect size was 0.61 (SMD = 0.61, 95% CI: 0.14–1.08), which was statistically significant (Z = 2.55, p = .01). In subgroup analysis, group-specific interventions, interventions for >4 weeks, and untreated control studies were more effective. Conclusion: This study confirms that nonpharmacological interventions are effective in improving sleep quality among the elderly in long-term care facilities. However, the small sample size and risk of bias in assessing the interventions of individual studies are unclear or high, thereby limiting the generalizability of the results. Further studies based on randomized control trials and the development of evidence-based interventions that consider the elderly participants’ physical activity levels, intervention methods and duration, and control group selection are needed to obtain more conclusive evidence.

